# Cellular and Molecular Mechanisms in Oxidative Stress-Related Diseases

**DOI:** 10.3390/ijms23148017

**Published:** 2022-07-20

**Authors:** Alessia Remigante, Rossana Morabito

**Affiliations:** Department of Chemical, Biological, Pharmaceutical and Environmental Sciences, University of Messina, 98122 Messina, Italy; rmorabito@unime.it

The redox equilibrium is important in preserving the correct functionality of vital cellular functions. Oxidative stress occurs in both nucleated and non-nucleated cells, and is defined as the imbalance in the redox characteristics of some cellular environments, which can be the result of biochemical processes leading to the production of reactive species, exposure to damaging agents (environmental pollutants and/or radiations), or the limited capabilities of endogenous antioxidant machinery. In these conditions, all cellular biomolecules can be altered through oxidation to an extent that exceeds the cell’s repair capacity [1,2]. Thus, several defense systems are involved within the cells to prevent an uncontrolled increase in reactive oxygen and nitrogen species (ROS/RNS). These systems include non-enzymatic molecules (glutathione, vitamins A, C, and E, and several anti-oxidants present in foods) as well as enzymatic scavengers of ROS, such as superoxide dismutase, catalase, and glutathione peroxidase, being the best-known defense systems [3]. During the past decade, research has revealed the widespread involvement of oxidative stress in a number of disease processes, including pulmonary and liver diseases, cancer, and ocular degenerative diseases. Moreover, these pathologic states have increasing incidence with age, and oxidative stress is believed to be a major factor in aging and aging-associated diseases. Thus, oxidative stress markers are important tools to assess the biological redox status, disease state and progression, as well as the health-enhancing effects of antioxidants in humans [4]. The aim of this Special Issue was to collect and contribute to the dissemination of high-quality research articles, as well as review articles, focusing on the relationship between oxidative-stress-related diseases and cellular responses in different pathologies. In addition, molecular targets of the cellular membrane, as well as their potential modulation under oxidative stress, were also considered, in an attempt to provide more information about the cell response to oxidative stress and its possible modulation using natural antioxidants. Here, we offer an overview of the content of this Special Issue, which collects four reviews and three original articles.

Exposure of the airway epithelium to environmental insults, including cigarette smoke, results in increased oxidative stress due to an imbalance between oxidants and antioxidants in favor of oxidants. In fact, the bronchial epithelium constitutes the front line in the defense of the human lung against invading pathogens and inhaled toxic agents. Bronchial epithelial cells are therefore well equipped with robust and redundant antioxidant and detoxifying systems to counteract insults derived from the external environment. Nevertheless, when the exposure to oxidants is very strong and prolonged over time, endogenous antioxidant systems are overwhelmed and can no longer maintain cellular homeostasis. This generates a condition known as oxidative stress, where a number of pro-inflammatory pathways become activated, generating a feed-forward loop that is difficult to resolve and that most often results in chronic inflammation and tissue injury. Cigarette smoke (CS) represents a major risk factor for most chronic inflammatory diseases, especially lung diseases, such as obstructive pulmonary disease. The use of experimental models representative of the lung epithelium insulted by CS provides the tools for the study of the molecular mechanisms underlying oxidative-stress-related diseases, in order to discover new therapeutic targets and develop new drugs. Promoting endogenous antioxidant systems, as well as relieving oxidative insults through natural or synthetic antioxidant compounds, may be useful strategies for the development of new therapies. In this scenario, the possibility of the real-time assessment of oxidative stress in readily available biological fluids is becoming of great importance to monitor disease progression and therapeutic response. In this respect, the development of innovative electrochemical sensor-based detection systems is paving the way for improved diagnosis, telemedicine and targeted therapeutic approaches. Stopping or avoiding smoking cigarettes, a good nutritional status with a proper intake of natural antioxidants, and regular physical activity remain the three fundamental factors to counteract oxidative stress and mitochondrial damage and represent the best primary prevention strategy to preserve human health [5]. Oxidative stress also contributes to the initiation and progression of liver injury. Many risk factors, including alcohol, may induce oxidative stress in the liver, which in turn results in severe liver diseases, such as alcoholic liver disease (ALD) [6]. The accumulation of clinically relevant knowledge regarding the role of oxidative stress and inflammation will help develop optimal experimental ALD models, which will facilitate the rapid screening and pharmacological study of potential therapeutic agents. Although no approved medications for ALD have been developed based on a strategy specifically targeting oxidative stress, recent clinical trials suggest that antioxidant drugs or drugs inhibiting inflammatory liver injury may be used to treat patients with ALD in the future.

Generally, ROS are continuously generated in the human body, although ROS-scavenging systems eliminate unnecessary ROS to preserve redox homeostasis. In the case of abnormal ROS production or the malfunction of antioxidant systems, redox imbalance can occur, which promotes the initiation and progression of several types of cancer. It has been well established that cancer cells are under constant oxidative stress, as reflected by an elevated basal level of ROS, due to increased metabolism driven by aberrant cell growth. The down-regulation of ROS facilitates pre-cancerous tumor development, even though increasing the level of ROS can promote metastasis. The transforming growth factor-beta (TGF-β) signaling pathway plays an anti-tumorigenic role in the initial stages of cancer development, but a pro-tumorigenic role in later stages that fosters cancer metastasis. Consequently, these events influence the potential for utilizing ROS as a therapeutic target [7]. Crosstalk among oxidative stress/ROS and TGF-β occurs in cancer cells, i.e., TGF-β controls oxidative stress by both boosting ROS production and controlling the antioxidant systems, whereas ROS control the TGF-β signaling pathway, encouraging cancer invasiveness. Therefore, as the ROS levels are higher in cancer cells than normal cells, cancer cells might be more vulnerable than normal cells to an increase in ROS, thus providing a therapeutic opportunity. Explaining the complicated relationship and functions of TGF-β and oxidative stress in cancer is significant for identifying their involvement in the initiation, development, and metastasis of tumors, and could ultimately reveal possible combinatory therapeutics for upcoming tests for cancer in humans. Thus, the inhibition or control of the amplification loop between TGF-β and the ROS system in cancer cells could reduce tumor development and metastasis, weakening tumor dissemination, proliferation, and survival. In this regard, the tumor microenvironment is often characterized by an increase in oxidative stress levels [8]. When oxidants are produced in excess, or when the antioxidant defenses are ineffective, biomolecules can be damaged. In this regard, ion channels possess sulfur-containing cysteine and methionine residues that can be targeted by ROS, thus altering channel function, including gating and conductivity properties, as well as associated signaling pathways. Specifically, the amino acid methionine is particularly susceptible to oxidation due to the presence of a highly reactive sulfur atom within a hydrophobic side chain. In fact, in oxidative conditions, methionine is oxidized to methionine sulfoxide (met-O), thus altering the secondary structure of the target protein. Among oxidant agents, chloramine-T (Chl-T) efficiently oxidizes methionine, and represents an ideal tool to mimic abnormal intracellular ROS levels. Ferrera and collaborators studied the response to oxidative stimulation in human primary (IGR39) or metastatic (IGR37) cell lines obtained from the same patient [9]. In IGR39 cells, Chl-T activated large K^+^ currents (KROS), which were partially sensitive to tetraethylammonium (TEA). A large fraction of KROS was inhibited by paxilline, a specific inhibitor of large-conductance Ca^2+^-activated BK channels. The TEA-insensitive component was inhibited by senicapoc, a specific inhibitor of the Ca^2+^-activated KCa3.1 channel. Both BK and KCa3.1 activation were mediated by an increase in [Ca^2+^]i induced by Chl-T. The increase in both KROS and [Ca^2+^]i was inhibited by ACA and clotrimazole, two different inhibitors of the calcium-permeable TRPM2 channel. Surprisingly, IGR37 cells did not exhibit a current increase upon the application of Chl-T. Expression analysis confirmed that the genes encoding BK, KCa3.1, and TRPM2 are much more expressed in IGR39 than in IGR37. The potassium currents and [Ca^2+^]i increase observed in response to the oxidizing agent strongly suggest that these three molecular entities play a major role in the progression of melanoma. The pharmacological targeting of either of these ion channels could be a new strategy to reduce the metastatic potential of melanoma cells, and could complement classical radio or chemotherapeutic treatments.

Since the theory of aging being caused by free radicals was published in 1956, various studies have gradually confirmed the dysregulation of oxidative stress as a critical precipitating or exacerbating factor in many pathological processes and the development of diseases. Aging is a dynamic chronological process characterized by the gradual accumulation of damage to cells, progressive functional decline, and increased susceptibility to disease [10]. These oxidative-stress-related diseases include numerous ocular degenerative diseases [11]. As the main photosensitive organ, the eye directly receives the energy found in sunlight, which travels through the cornea, anterior chamber, lens, and vitreous body to the retina. In addition to causing DNA damage, ultraviolet (UV) light can also cause photo-oxidative stress through the production of ROS. ROS lead to cell damage and aging, resulting in corneal degeneration, lens opacification (cataracts), and the occurrence of eye diseases, including various retinal and optic nerve degenerative diseases, such as glaucoma and age-related macular degeneration (AMD). Using antioxidant biomarkers, patients with a low antioxidant capacity can be identified, and antioxidant supplementation can be used for disease prevention, delay, or treatment.

Natural compounds counteract the formation of ROS, which are normally produced as by-products of the cellular metabolism or are induced by extracellular stimuli. For example, flavonoids are a class of naturally occurring polyphenolic compounds and are important antioxidants and free radical scavengers characterized by high biochemical activity and a variety of pharmacological effects. In particular, quercetin (3,5,7,3′,4′-pentahydroxyflavone; Q) is part of a group of bioflavonoids that are naturally occurring in fruits and vegetables. This molecule can directly neutralize ROS/RNS and/or inactivate molecules with pro-oxidant capacity. Remigante and co-authors demonstrated that the stability of the erythrocyte membranes can be positively affected by exogenous antioxidants, such as Q [12]. In particular, the purpose of the study was to investigate the protective role of Q in a D-galactose (D-Gal)-induced model of aging in human erythrocytes. The results confirm that D-Gal activated oxidative stress pathways in human erythrocytes, affecting both membrane lipids and proteins, as denoted by increased TBARS levels and decreased total sulfhydryl groups, respectively. In addition, D-Gal led to an acceleration of the rate constant of the SO_4_^2−^ uptake through the Band 3 protein (B3p). Both the alteration of the B3p function and oxidative damage were improved by pre-treatment with Q, which preferentially ameliorated lipid peroxidation rather than protein oxidation. Moreover, Q prevented glycated A1c formation, while no protective effect on the endogenous antioxidant system was observed. These findings suggest that the B3p could be a novel potential target of antioxidant treatments to counteract aging-related disturbances. Further studies are needed to confirm the possible role of Q in pharmacological strategies against aging.

In conclusion, the redox equilibrium plays a critical role in ischemia/reperfusion injury. Mesenteric ischemia and reperfusion (I/R) injury can ensue from a variety of vascular diseases and represents a major cause of morbidity and mortality in intensive care units. It causes an inflammatory response associated with local gut dysfunction and organ injury. Since ischemia is rarely preventable, most studies have concentrated on the strategy for the reperfusion period and the development of novel therapeutic approaches for reducing ROS, in order to limit the overloaded inflammatory response and attenuate cellular damage. In this regard, adenosine monophosphate-activated protein kinase (AMPK) is a crucial regulator of metabolic homeostasis. The catalytic α1 subunit is highly expressed in the intestine and vascular system. In loss-of-function studies, the biological role of AMPKα1 in the function of the gastrointestinal barrier was investigated by Hayes and collaborators [13]. Male knock-out (KO) mice with a systemic deficiency of AMPKα1 and wild-type (WT) mice were subjected to a 30 min occlusion of the superior mesenteric artery. Four hours after reperfusion, AMPKα1 KO mice exhibited exaggerated histological gut injury and the impairment of intestinal permeability associated with marked tissue lipid peroxidation, and a lower apical expression of the junction proteins occludin and E-cadherin when compared to WT mice. Lung injury with neutrophil sequestration was higher in AMPKα1 KO mice than WT mice and paralleled higher plasma levels of syndecan-1, a biomarker of endothelial injury. Thus, the data demonstrate that AMPKα1 is an important requisite for epithelial and endothelial integrity and plays a protective role in remote organ injury after acute ischemic events.
